# Green credit and enterprise green operation: Based on the perspective of enterprise green transformation

**DOI:** 10.3389/fpsyg.2022.1041798

**Published:** 2022-10-05

**Authors:** Haiyan Niu, Xiongfei Zhao, Zhilin Luo, Yuxia Gong, Xinhua Zhang

**Affiliations:** ^1^College of Economics and Management, Tianjin University of Science and Technology, Tianjin, China; ^2^School of Economics and Management, Beijing University of Technology, Beijing, China; ^3^General Education Faculty, Chongqing Industry Polytechnic College, Chongqing, China; ^4^Suzhou Early Childhood Education College, Suzhou, China

**Keywords:** green credit policy, green operation, heavy pollution enterprises, debt costs, government subsidies

## Abstract

This paper uses panel data of listed heavily polluting enterprises from 2007 to 2021, based on the perspective of transformation and upgrading of heavy polluters, innovatively studies the impact of green credit on the green operation of enterprises. At the micro level, the research results of this paper verify the effectiveness of green credit policy on the transformation of green enterprises. It is also found that the two intermediary paths of debt cost and government subsidy play a partial intermediary role in the process of green credit promoting green enterprise transformation and upgrading. Green credit policy also moderates the green transformation of enterprises through debt cost and government subsidies. Based on the research results, this paper puts forward targeted policy suggestions from the aspects of financing constraints, government subsidy policies, enterprise technological innovation and green operation, and provides empirical support for the current expansion of green credit policies in China.

## Introduction

Rapid urbanization and industrialization have caused serious environmental pollution, which has posed serious challenges to ecosystems and economic development. Climate change has become a widespread concern in the world today. The Chinese economy has shifted from high-speed growth to high-quality development, and environmental pollution has become one of the major factors limiting China’s economy’s high-quality development (Zhao and Luo, 2018), and achieving coordinated ecological protection and economic growth is an inherent requirement for high-quality economic development (Xing et al., 2019). To reduce pollution emissions and support high-quality economic development, the Chinese government has implemented a number of green financial policies (An et al., 2021; Lv et al., 2021; Lee and Lee, 2022), and green credit is a key component of green finance (Lian Y. et al., 2022). From the perspective of commercial banks, green credit incorporates enterprise environmental information disclosure into credit fund approval (Li et al., 2022), implementing resource allocation (He et al., 2019a). Correspondingly, from firms’ perspective, after the implementation of the green credit policy, it is more difficult for highly polluting enterprises to obtain loan financing, while green environmental protection enterprises are more likely to obtain bank credit funds (Yuan and Gao, 2022). Enterprises, as an important subject of economic development, the main means by which the idea of green development is put into practice and green transformation and upgrading is realized by businesses. This is also a key step in China’s economy’s transition to high-quality development (Ding et al., 2022a). Green development has become a new driving force for the green transformation and upgrading of enterprises (Farooq et al., 2021). Leveraging green credit funds to improve operational efficiency is key for companies to achieve growth and development and maintain a competitive advantage. By the end of 2020, the green credit balance of 21 major banks in China had exceeded 11 trillion yuan, and in terms of the proportion of credit funds to the total investment in green projects, the green qualifications of 21 major banks could support the saving of more than 300 million tons of standard coal and the reduction of more than 600 million carbon dioxide equivalent per year (Chai et al., 2022). With the gradual development of green credit policy, it is critical to examine the current Chinese green credit policy from the standpoint of enterprises in order to encourage heavily polluting companies to implement green innovation in order to transform and upgrade. This can lead to new concepts for enterprise green development as well as theoretical support for China’s green credit policy’s aggressive promotion in order to achieve sustainable development.

The implementation of green credit policy has had a significant impact on the development of highly polluting businesses, and many academics’ research has gradually shifted its focus to the relationship between green credit and businesses. However, there remain certain gaps in the literature. First, according to some academic studies on corporate financing, green credit policy restricts firms’ debt and equity financing, and its impact is greater on state-owned enterprises and regions with fragile financial ecosystems (Tian et al., 2022). Only green innovation could make it easier for businesses to obtain loans, and companies that disclosed more about their environmental practices did not receive more loans (Xing et al., 2021). Second, in terms of investment efficiency, Green credit policies have a double threshold effect on renewable energy investments, with the effect divided into three stages: promotion, inhibition, and facilitation (He et al., 2019b). This can improve the effectiveness of enterprises’ overseas investment, particularly for state-owned enterprises and enterprises in environmentally lax regions (Zhang J. et al., 2022). Furthermore, green credit policies reduce the level of policy investment (Zhang, 2022). However, very limited attention is given to green transformation and upgrading of enterprises. Thus, further research is urgently needed.

This paper’s potential contributions to existing research are primarily reflected in the following aspects: First, different from the previous research on green transformation and upgrading of enterprises from the perspective of financial development and green finance, this paper innovatively incorporates green credit into the research scope, which makes the research perspective more refined. Based on the analysis of the theoretical mechanism, this paper empirically tests the impact of green credit on the green transformation and upgrading of enterprises, deepens the research on the application of green finance in the ecological effect of enterprises, and greatly fill the gap of theoretical studies on green credit policies. Second, unlike previous static studies, this paper develops a mediation effect model to test the dynamic trajectory of green credit policies influencing enterprises’ green transformation, validates the possible mediation transmission mechanism between variables, and tries to deconstruct the inner logic of green credit and enterprise transformation and upgrading, further to enrich the empirical study of green credit policies. Third, we further investigate whether green credit promotes or inhibits the process of enterprise transformation under the influence of adjustment variables, which will deepen the theoretical research and guide policy practice for bank credit to influence the transformation and upgrading of enterprises.

The overall layout of the article is as follows: the second part conducts literature review; the third part conducts theoretical analysis and research hypotheses; the fourth part conducts research design and statistical analysis; the fifth part analyzes experimental results; and finally summarizes the conclusions and makes policy recommendations.

## Literature review

Green credit policy is a broad environmental regulation based on the theory of environmental economics, Some academics are looking into how green credit policies affect corporate green innovation (Huergo and Moreno, 2017; Cao and Leung, 2020; Chiu and Lee, 2020; Männasoo and Meriküll, 2020). At this stage, there are two main theories on the relationship between environmental regulation and green innovation: Pollution refuge hypothesis (Peng, 2020) and Porter hypothesis (Rubashkina et al., 2015). Pollution refuge hypothesis stems from compliance cost, arguing strict environmental regulations can increase firms’ production costs, and therefore discourage innovation (Ouyang et al., 2020). Porter hypothesis suggests that appropriate environmental regulations can improve firm productivity and facilitates firm technological innovation (Hashmi and Alam, 2019). Based on the above theory, existing studies have examined the impact of environmental regulations on green transformation from different perspectives. Properly crafted environmental regulatory policies can facilitate the process of technological innovation (Liu et al., 2022), resulting in compensation effects for innovation and fostering green innovation (Tang et al., 2020), which verifies the Porter hypothesis. Green industrial policies can stimulate corporate green innovation through the intermediary role of government subsidies and bank loans, with the promotion effect being more pronounced for state-owned enterprises (Zhu and Tan, 2022). Green credit can also stimulate corporate green innovation through financing constraints, with the stimulus being more pronounced among firms with high expected sunk costs or non-compliance costs, competitive advantages, and competitive advantages (Hu et al., 2021). Green credit policies boost corporate investment in research and development and management effectiveness to encourage corporate innovation in low-carbon technologies (Chen et al., 2022). Agency costs have a negative impact on green credit’s ability to foster technological innovation, while profitability and equity concentration have positive effects (Zhang S. et al., 2022). Green credit policies enhance overall and incremental green innovation (Kong et al., 2022; Zhang Y. et al., 2022), but they discourage aggressive green innovation in high polluting firms; they also enhance innovation quality (Wang et al., 2022) and can further increase firms’ product competitiveness. Businesses with weak environmental compliance laws will use technological innovation more effectively once the Green Credit Guidelines are in place, according to the impact of the Green Credit Guidelines’ implementation on green innovation (Wang and Li, 2022). Green credit policies, according to one point of view, will have a negative impact on the performance of businesses operating in highly polluting industries. During periods of high economic and state-owned enterprise policy uncertainty, this effect will be more pronounced (Yao et al., 2021; Tan et al., 2022). Determining the precise role of green credit in the green transformation of enterprises is thus critical for promoting the growth of high-quality economies.

The focus of promoting high-quality development in China’s economy is on industrial structure optimization, as well as green transformation and enterprise upgrading. China’s economy is at a crossroads in terms of quality development. Green credit policies increase the tendency of heavy polluters to diversify, and this effect is more pronounced for large-scale and poorly governed heavy polluters. Green credit policies also help to modernize and upgrade polluting industries (Li and Chen, 2022). Green credit optimizes and upgrades the industrial structure by promoting the transformation and upgrading of highly polluting enterprises by establishing green financial instruments and assisting businesses in the development of new energy-saving and environmental protection technologies (Anderson, 2016). Through a series of green financial financing, green finance can promote technological progress and upgrade industrial structure (Song et al., 2022). When looking at green development through the lens of spatial spillover effects, the synergistic industrial agglomeration plays a positive role in promoting regional green development by generating spatial agglomeration and spatial spillover effects, and the intermediary role of the level of green technological progress is more prominent in this process (Ding et al., 2022a). The findings of a study of industrial structure upgrading from the perspective of a low-carbon city pilot show that the initiative can support it by fostering technological innovation and lowering the share of high-carbon industries (Zheng et al., 2021). Government macro-control works to transform and upgrade industrial results by improving environmental regulation policy tools, pursuing precise environmental regulation, developing an environmental information service platform, encouraging support for reforms in critical areas, and strengthening environmental regulation (Yu and Wang, 2021).

In summary, the existing literature on green credit policies provides a useful reference for the authors’ study and provides a theoretical basis for the authors to continue their in-depth study of the relationship between green credit and corporate green operations. However, there are two shortcomings in the existing studies: First, there is still not enough research on theoretical analysis, empirical studies and policy effects related to green credit and the effect of green credit policy implementation still needs to be further explored. Second, the transmission mechanism of green credit policy is still not perfect. Although the literature has revealed the effects of green credit implementation from different perspectives, less literature has studied the relationship between green credit and green operations of enterprises, which provides a creative perspective for the authors. As an important financial instrument to support green and low-carbon development, an in-depth discussion on the induced mechanism of green credit policies affecting green operations of enterprises can provide rich policy recommendations for achieving green and low-carbon development.

## Theoretical analysis and research hypothesis

### The impact of green credit policy on the green transformation and upgrading of enterprises

Green credit policy promotes green transformation and upgrading of businesses by differentiating credit rates through “incentive” and “push” mechanisms (Nandy and Lodh, 2012). First, the green credit policy directs banking financial institutions to allocate credit resources and capital formation mechanisms in favor of low-pollution, low-consumption, energy-saving, and environmentally friendly industries, while increasing credit costs or discontinuing lending to the original high pollution and high energy-consuming enterprises (Chang et al., 2019; Wen et al., 2021). Due to the differentiated green credit policy, the high pollution and high energy-consuming polluting enterprises’ ability to obtain financing is limited, and the scale of enterprise financing is severely constrained, forcing enterprises to undergo a green transformation. Green development enterprises that benefit from preferential interest rates can obtain capital from financial institutions to supplement their development capital and encourage them to continue developing their own energy-saving and environmental protection projects while also maximizing other types of projects and production. Green credit policies, according to Luo et al. (2021), can have an impact on bank performance and competitiveness. Commercial banks’ credit risk is reduced and their reputation is enhanced by encouraging green credit (Scholtens and Dam, 2007), which increases banks’ cost effectiveness (Chen et al., 2018). As a result, green credit policies can, to some extent, restrain the blind and disorderly expansion of high-energy-consuming (Xu and Li, 2020), high-polluting enterprises while also promoting sustainable development. Banks are more willing to provide adequate funding for enterprises with high technological content and low pollution emissions, reducing the source of credit for energy-intensive industries and promoting the development of environmentally friendly industries. Accordingly, this paper proposes hypothesis 1:

*Hypothesis 1: Green credit policies can promote the transformation and upgrading of heavy polluters.*

### The mediating role of debt costs

Green credit policy influences business production and operation through financing costs, resource allocation, and market orientation. Green credit is a special fund provided by financial institutions to promote corporate green development, and the high cost of debt has a direct impact on corporate debt financing. Environmental protection is also incorporated into financial institutions’ daily business philosophies, with full consideration given to environmental risks and potential rewards (Zhang et al., 2011; Nobanee and Ellili, 2016; Yin et al., 2021). Financial institutions seek to maximize their own financial interests while also seeking to maximize environmental benefits, fully acknowledging the critical role that banking and financial institutions play in environmental protection. On the one hand, the green credit policy has raised the credit threshold for heavily polluting enterprises, resulting in a significant financing penalty for these businesses (Lu et al., 2022). Therefore, the financing costs of heavily polluting enterprises have risen, forcing them to focus more on transformation and upgrading. Green credit policy, on the other hand, can direct the flow of funds to enterprise environmental protection projects, and production resources are gradually shifting toward green enterprises (Liu et al., 2017; Xing et al., 2020). Furthermore, according to signal theory, green credit policies strengthen the concept of social green development gains traction, the public’s pressure on polluting businesses grows companies take more social responsibility for environmental protection, creditors and investors decide whether to finance based on the company’s environmental performance (Li et al., 2020). Green finance will improve the channels for environmental information disclosure at the same time, allowing the general public and investors to obtain more detailed information on enterprise projects while also encouraging green transformation and upgradation of businesses (Fuente et al., 2017). Therefore, we propose the following hypothesis 2:

*Hypothesis 2: Green credit policy raises the cost of debt for heavy polluters, which in turn promotes green transformation and upgrading of enterprises.*

### The mediating role of government subsidies

Government subsidies are an important tool for addressing market failures and for achieving macroeconomic regulation, optimizing social resource distribution, and ensuring the smooth operation of the market economic system (Peng and Liu, 2018). Furthermore, they act as an external incentive to address the issue of externality and encourage businesses to engage in green technology innovation activities (Kang and Park, 2012; Dai and Cheng, 2015; Bronzini and Piselli, 2016). According to signaling theory, information asymmetry between businesses and the government can result in a “horse-trading effect” in government subsidies, leading to a “free-rider” phenomenon for quasi-public goods like environmental resources (Antonelli and Crespi, 2013). In other words, government subsidies tend to favor green businesses when implementing green credit policy. From the perspective of local governments, green development enterprises assume more environmental responsibilities, which contributes to the improvement of local governments’ environmental performance; from the perspective of enterprises, green development enterprises are proactive in technological innovation while assisting governments in achieving environmental performance (Li et al., 2018), bringing government-enterprise relations closer together (Kostovetsky, 2015; Chen et al., 2020). With regard to the enterprises’ own goals for obtaining government grants, the effect of grants on the green transformation of businesses can vary greatly. If businesses use green development as a way to obtain government subsidies, their green activities are essentially commercial interests, forming unfavorable selection of subsidies from the government and encouraging rent-seeking behavior from subsidies (Zhong et al., 2022). However, when businesses uphold their social obligations and include green development in their sustainable development strategy, government subsidies can quickly ease the plight of businesses when heavy polluters are short on funds (Huang et al., 2019) and promote green innovation (Song et al., 2020). At the same time, guided by green credit policies, it can release positive signals to the outside world and improve the financing ability of heavy polluters (Ren et al., 2020). Government subsidies will therefore be more likely to go to green development enterprises in the context of the country’s vigorous implementation of green credit policies, improving the effectiveness of local environmental governance and, to some extent, avoiding the risk of subsidies. Accordingly, this paper proposes hypothesis 3:

*Hypothesis 3: Green credit policies can increase government subsidies for heavy polluters, which in turn will promote green transformation and upgrading of enterprises.*

## Research design

### Variable selection

#### Dependent variable: Total factor productivity of enterprises

This paper mainly studies the influence of green credit policy on the green transformation and upgrading of heavy pollution enterprises, and the green transformation and upgrading of enterprises is the dependent variable of this paper. Some scholars (Shi and Li, 2019; Wu et al., 2020; Xia and Xu, 2020) study green total factor productivity (*Gtfp*), but all focus on the macro level and green innovation (Wu and Zhang, 2020; Fang et al., 2021; Wang et al., 2021). *Gtfp* integrated assessment of changes in social welfare and economic development performance (Cao et al., 2021). But the transformation and upgrading of enterprises is ultimately reflected in the productivity of enterprises, and the manifestation of transformation and upgrading may differ from one enterprise to another, however, it is mainly manifested in the overall improvement of production technology, resource allocation and a series of investment activities of enterprises (Yoruk, 2019). Therefore, this paper uses total factor productivity (*Tfp*) as the dependent variable. The main reasons for the differences in the judgments of *Tfp* or *Gtfp* are: First, the choice and treatment of indicators imply different systems of input-output indicators, which can lead to different accounting results. Second, different rates and patterns of economic growth in different periods lead to different results (Xu and Deng, 2022). The calculation of *Tfp* can be traced back to the Solow residuals in the Solow model, which is the growth rate of output minus the growth rate of factor inputs (Wu et al., 2022). For inputs and outputs, *Tfp* can be calculated using capital, labor, intermediate inputs, and gross output (Li and Lin, 2016; Zhang and Du, 2020). *Tpf* measurement methods include: OLS method, OP method, LP method, etc. (Ai et al., 2020). Currently, the OP method is widely used, which is based on a semi-parametric estimation method that minimizes the endogeneity problem. However, the OP method has some drawbacks, for example, due to the use of intermediate investment as a proxy variable, which makes the estimates not take into account the relationship between labor input and residuals, etc. Therefore, the LP estimation method is proposed. This paper uses the LP method to measure the total factor productivity of enterprises (Liu et al., 2021; Nakatani, 2021), and the total factor productivity calculated by the OP method is then used to test the robustness of the model. The measurement formula is as follows:


(1) $$LnY_{i.t}=\text{α}+\text{β}LnL_{i.t}+\text{γ}LnK_{i.t}+\text{δ}M_{i.t}+{\text{ε}}_{i.t}$$

Where Output (*Y*_*i.t*_) is the Operating income of company *i* in year *t*; input are *L_i.t_* and *K*_*i.t*_, *whichL*_*i*.*t*_ is the number of employees of firm *i* in year *t* and *K*_*i.t*_ is the net value of fixed assets of firm *i* in year *t*; the intermediate inputs (*M*_*i.t*_) are cash paid by company *i* for goods purchased and services received in year *t*; and ε_*i*.*t*_ is total factor productivity (*Tfp*).

#### Explanatory variables: Green credit policy

This paper constructs green credit indicators through policy dummy variables. In 2012, the CBRC issued the Green Credit Guidelines, which formally introduced the green credit policy (Liu et al., 2019), therefore, this paper takes 2012 as the time point and sets it as 0 before 2012 and 1 for 2012 and beyond.

#### Intermediate variables

(1) Cost of debt (*Cdf*): Since the interest levels of a firm’s short-term and long-term borrowings are not disclosed separately in the firm’s financial reports, it seems impossible to obtain the exact cost of each. Therefore, we define the cost of debt (*Cdf*) as the ratio of interest expense to total debt for listed companies (Xu and Li, 2020). (2) Government subsidies (*Subs*): According to the time sequence of government subsidies and corporate R&D, government subsidies can be divided into: government subsidies beforehand (CSB) and government subsidies afterward (Hud and Hussinger, 2015), We focus primarily on government subsidies beforehand, therefore using the logarithm of the government subsidies received by the firm during the year to measure government subsidies (Lian G. et al., 2022).

#### Control variables

In the analysis of the green transformation and upgrading of enterprises, other factors will have an impact on the effect of green transformation of enterprises. In order to reduce the bias of omitted explanatory variables on the regression estimation results and to make the model more representative, referring to existing research (Hu et al., 2021; Meng and Zhang, 2022; Shi et al., 2022), the following control variables are included in the model: (1) Age of listing (*Age*): expressed in terms of the time that companies have been listed as of now; (2) Cash ratio (*Cash*): expressed as a ratio of cash and cash equivalents to current assets; (3) Return on total assets (*Roa*): expressed as the ratio of EBITDA to total assets; (4) Net profit margin on total assets (*Roe*): Expressed as the ratio of net profit to average total assets; (5) Gearing ratio (*Lev*): expressed as the ratio of total liabilities to total assets at the end of the year; (6) Tangible assets ratio (*Tang*): expressed as the ratio of total fixed assets to total assets at the end of the year.

### Data sources and descriptive statistics

The article selects the panel data of heavy polluting enterprises listed in Shenzhen and Shanghai A-shares from 2007 to 2021, and the heavy polluting enterprises are selected with reference to the 2012 edition of the industry classification of the Securities and Futures Commission, and in order to ensure the quality of the sample data, this paper treats the data as follows: (1) The data that were ST and *ST were excluded. (2) Data with serious missing data in the sample are excluded. (3) Excluded enterprises listed after 2012. Finally, 777 heavily polluting enterprises were selected, and the descriptive statistics of the variables are shown in Table 1. Considering that the high correlation of variables may cause the problem of multicollinearity, which affects the accuracy of the estimation results, this paper tests by variance inflation factor, and the results show that the VIF value is less than 10, so the model does not have serious multicollinearity problem, which ensures the relative reliability of the estimation results. The data used for the variables were obtained from the CSMAR and Wind databases.

**TABLE 1:** Descriptive statistics of variables.

	N	Mean	Median	Std. Dev.	Min	Max
*lnTfp*	7014	15.013	14.899	0.895	12.237	18.957
*GCL*	7014	0.627	1.000	0.484	0	1
*lnCdf*	7014	–2.864	–2.945	0.918	–10.659	3.024
*lnSubs*	7014	16.224	16.252	1.933	6.908	24.642
*lnAge*	7014	2.904	2.996	0.329	2.303	3.401
*lnCash*	7014	–1.025	–1.088	1.171	–5.537	5.121
*lnRoa*	7014	–2.811	–2.787	0.699	–12.914	–.422
*lnTang*	7014	–0.079	–0.048	0.099	–1.148	0
*lnLev*	7014	–0.961	–0.828	0.57	–4.95	0.037
*lnRoe*	7014	–3.386	–3.179	1.103	–9.641	–0.583

### Model construction

#### Baseline regression model

To test the direct impact of green credit policies on the green transformation of enterprises, the article constructs the following baseline regression model:


(2) $$lnTfp_{i.t}={\text{β}}_{0}+{\text{β}}_{1}GCL_{i.t}+{\text{β}}_{2}lnControls_{i.t}+\vartheta_{i}+\delta_{t}+\mu_{i.t}$$

Equation (2), where *i* represents the security code, *t* represents the year, and Controls represents the control variables. β_0_, β_1_, β_2_ are the coefficients to be estimated for the constant term, the explanatory variable *GCL*, and the control variable lnControls, respectively. ϑ_*i*_ denotes individual fixed effects, δ_*t*_ denotes time fixed effects, and μ_*i.t*_ is the random disturbance term. To eliminate the effect of heteroskedasticity, each variable was logarithmicized and denoted as *ln*.

#### Mediating effect model

This paper draws on (He et al., 2020) to develop an empirical model containing multiple mediating variables to test the specific path of green credit acting on firms’ green transformation and upgrading through cost of debt (*Cdf*) and government subsidies (*Subs*). On the basis of the baseline regression model, the model is set up as follows:


(3) $$lnCdf_{i.t}=a_{0}+a_{1}GCL_{i.t}+a_{2}lnControls_{i.t}+\mu_{i.t}$$

(4) $$lnSubs_{i.t}=b_{0}+b_{1}GCL_{i.t}+b_{2}lnControls_{i.t}+\mu_{i.t}$$

(5) $$lnTfp_{i.t}=c_{0}+c_{1}GCL_{i.t}+c_{2}lnCdf_{i.t}+c_{3}lnControls_{i.t}+\mu_{i.t}$$

(6) $$lnTfp_{i.t}=d_{0}+d_{1}GCL_{i.t}+d_{2}lnSubs_{i.t}+d_{3}lnControls_{i.t}+\mu_{i.t}$$

In Equations (3–6), *lnCdf* and *lnSubs* denote the two mediating variables of debt cost and government subsidy, respectively, and the meanings of other variables are consistent with those in model (1). Model (3) represents the effect of green credit policy on the cost of debt and the sign of the expected coefficient *a_1_* is positive, indicating that green credit policy can increase the cost of debt for firms. Model (4) represents the effect of green credit policy on government subsidies and the sign of the expected coefficient *b*_1_is positive, indicating that green credit policy increases the amount of government subsidies. Model (5) represents the effect of green credit policy on firms’ total factor productivity when controlling for the cost of debt. The coefficient *c*_1_ represents the direct effect of green credit policy on firms’ total factor productivity, and the product of coefficient *a_1_* and coefficient *c_2_* represents the mediating effect of the cost of debt. Model (6) represents the direct effect of green credit policy on firms’ total factor productivity as coefficient *d*_1_ when controlling for the government subsidy variable, and the product of coefficient *b*_1_ and coefficient *d*_2_ represents the mediating effect of government subsidy.

#### Moderating effect model


(7) $$lnTfp_{i.t}=e_{0}+e_{1}GCL_{i.t}+e_{2}lnGcl_{i.t}lnCdf_{i.t}+\mu_{i.t}$$

(8) $$lnTfp_{i.t}=f_{0}+f_{1}GCL_{i.t}+f_{2}lnGcl_{i.t}lnSubs_{i.t}+\mu_{i.t}$$

The *e_2_* and *f_2_* in Equations (7) and (8) are the estimated coefficients of the regression of the interaction term (moderating effect) of green credit policy *GCL* with lncdf and lnsubs, respectively, and the meanings of other variables are consistent with those in model (1).

## Results of the empirical analysis

### Baseline regression

In order to analyze the impact of green credit policy on the total factor productivity of firms, this paper uses fixed-effect model to perform a baseline regression test on model (2), the results are shown in Table 2. Studying the effect of a single variable *Gcl* on *lnTfp* in column (1), it can be seen that the coefficient of green credit is positive at 1% level of significance, indicating that green credit policy significantly increases the total factor productivity of firms. To avoid omitting important explanatory variables, control variables were added to regress model (2), and the results are shown in column (2) of Table 2. The coefficient of green credit policy is significantly positive at the 1% level, which strengthens the explanation of the sample data and indicates that the implementation of green credit policy can improve the total factor productivity of enterprises. Then, adding individual fixed effects and time fixed effects to the control variables, the coefficient of green credit policy in column (3) of Table 2 is significantly positive at the 1% level. This finding is consistent with (Cui et al., 2022), hypothesis 1 holds.

**TABLE 2:** Baseline regression results.

Variables	*lnTfp*	*lnTfp*	*lnTfp*
	(1)	(2)	(3)
*GCL*	0.252***	0.373***	0.851***
	(11.50)	(17.50)	(21.88)
*lnAge*		0.354***	–0.797**
		(10.92)	(–2.56)
*lnCash*		0.013	0.046***
		(1.00)	(4.29)
*lnRoa*		0.302***	0.250***
		(10.85)	(11.88)
*lnTang*		0.542***	0.079
		(5.42)	(0.77)
*lnLev*		0.469***	0.207***
		(17.52)	(8.27)
*lnRoe*		–0.016	–0.047***
		(–0.83)	(–3.35)
*Constant*	14.855***	15.055***	18.343***
	(857.43)	(129.71)	(18.88)
*Control*	No	Yes	Yes
*Year Effects*	No	No	Yes
*Individual Effects*	No	No	Yes
*Observations*	7,014	7,014	7,014
*R-squared*	0.019	0.171	0.713

t-statistics in parentheses, *** *p* < 0.01, ** *p* < 0.05, * *p* < 0.1.

### Robustness and endogeneity tests

#### Robustness tests

The above test initially argues the hypothesis proposed in this paper, but there are many factors affecting the green transformation of enterprises, which leads to the problem of omitting variables in the above results to some extent. In order to avoid the bias caused by the chance of variables and make the research results more empirically valuable, the following methods are conducted in this paper to enhance the robustness of the empirical results:

First, substitution of explanatory variables. The transformation and upgrading of enterprises is not only reflected in the level of productivity, but also has an impact on R&D investment. Accordingly, this paper uses the firm’s R&D investment (*RD*) as the explanatory variable to re-estimates the Equation (2), and the regression results are shown in column (1) to (3) of Table 3, where (1) and (2) are regressed using OLS and (3) is using fixed effects model. It can be seen that the regression coefficient of green credit are all significantly positive at the 1% level, confirming the robustness of the above regression results.

**TABLE 3:** Robustness tests I: substitution of explanatory variables.

Variables	*RD*	*RD*	*RD*
	(1)	(2)	(3)
*GCL*	2.134***	1.937***	2.735***
	(36.53)	(33.96)	(23.90)
*lnAge*		–1.744***	2.729***
		(–20.10)	(2.98)
*lnCash*		0.081**	0.073**
		(2.38)	(2.33)
*lnRoa*		–0.118	–0.135**
		(–1.58)	(–2.18)
*lnTang*		0.244	0.211
		(0.91)	(0.70)
*lnLev*		–0.748***	–0.211***
		(–10.43)	(–2.86)
*lnRoe*		0.139***	0.050
		(2.77)	(1.21)
*Control*	No	Yes	Yes
*Year Effects*	No	No	Yes
*Individual Effects*	No	No	Yes
*Observations*	7,014	7,014	7,014
*R-squared*	0.160	0.286	0.701

t-statistics in parentheses, *** *p* < 0.01, ** *p* < 0.05, * *p* < 0.1.

Second, replacement measurement method. As we have analyzed in section “Variable selection,” this paper uses the OP method and the GMM method (Blundell and Bond, 1998) to measure total factor productivity to replace the results of the LP method used above for robustness testing, and the regression results are shown in column (1) to (6) of Table 4, and the regression coefficient of green credit are still significantly positive at the 1% level, confirming that the regression results above are more reliable.

**TABLE 4:** Robustness tests II: OP & GMM.

Variables	OP			GMM		
	*lnTpf*	*lnTpf*	*lnTpf*	*lnTpf*	*lnTpf*	*lnTpf*
	(1)	(2)	(3)	(4)	(5)	(6)
*GCL*	0.008***	0.016***	0.045***	0.008***	0.016***	0.045***
	(4.98)	(9.44)	(13.91)	(4.56)	(8.99)	(13.42)
*lnAge*		–0.001	–0.078***		–0.002	–0.082***
		(–0.27)	(–2.99)		(–0.81)	(–3.03)
*lnCash*		0.008***	0.012***		0.009***	0.012***
		(7.75)	(12.97)		(8.49)	(13.45)
*lnRoa*		0.022***	0.022***		0.022***	0.023***
		(10.08)	(12.36)		(9.79)	(12.31)
*lnTang*		0.059***	0.016*		0.057***	0.014
		(7.50)	(1.88)		(7.06)	(1.61)
*lnLev*		0.032***	0.000		0.033***	–0.000
		(15.08)	(0.02)		(15.02)	(–0.01)
*lnRoe*		–0.002	–0.005***		–0.001	–0.005***
		(–1.49)	(–4.18)		(–0.80)	(–3.73)
*Constant*	2.735***	2.829***	3.056***	2.634***	2.739***	2.967***
	(2,110.33)	(311.73)	(37.38)	(1,954.20)	(290.00)	(35.03)
*Control*	No	Yes	Yes	No	Yes	Yes
*Year Effects*	No	No	Yes	No	No	Yes
*Individual Effects*	No	No	Yes	No	No	Yes
*Observations*	7,014	7,014	7,014	7,014	7,014	7,014
*R-squared*	0.004	0.081	0.631	0.003	0.079	0.634

t-statistics in parentheses, *** *p* < 0.01, ** *p* < 0.05, * *p* < 0.1.

#### Endogeneity tests

There may be some indicators that are more difficult to quantify in studying the total factor productivity of enterprises, which will make the selected variables less comprehensive, and the above regression results may have endogeneity problems. For this reason, this paper uses green credit with a one-period lag as the instrumental variable and estimates it by two-stage least squares. Column (1) of Table 5 shows the results of the first-stage regression, where the estimated coefficient of the instrumental variable (*IV*) is 0.816 and significant at the 1% level, and the value of the F-statistic is much greater than 10, indicating that there is no weak instrumental variable problem. The results of the second stage of the regression are shown in column (2) of Table 5, where the estimated coefficient of green credit (*GCL*) is 0.581 and significant at the 1% level, which still supports that green credit can significantly increase the total factor productivity of heavily polluting firms, further confirming the robustness of the above regression results.

**TABLE 5:** Instrumental variables method regression results.

Variables	*lnTfp*	*lnTfp*
	(1)	(2)
*IV(L.GCL)*	0.816***	
	(117.26)	
*GCL*		0.581***
		(21.46)
*lnAge*	–0.087***	0.578***
	(–8.09)	(16.72)
*lnCash*	–0.002	–0.017
	(–0.50)	(–1.21)
*lnRoa*	0.002	0.283***
	(0.17)	(9.10)
*lnTang*	–0.048	0.419***
	(–1.46)	(4.05)
*lnLev*	0.003	0.498***
	(0.30)	(16.84)
*lnRoe*	–0.010	–0.010
	(–1.51)	(–0.47)
*Constant*	0.398***	14.220***
	(10.09)	(111.51)
*Observations*	5,341	5,341
*R-squared*	0.749	0.227

t-statistics in parentheses, *** *p* < 0.01, ** *p* < 0.05, * *p* < 0.1.

### Quantile regression

We examine the average marginal effect of green credit policy on heavy polluting firms’ total factor productivity in Table 2. In order to capture the variation of total factor productivity among different heavy polluting firms, four quartiles of 0.1, 0.25, 0.5, and 0.75 were chosen for estimation in this paper, Table 6 shows the results, and additional quantile regression models were developed to explain the variability. To begin with, the coefficients for green credit pass the significance test at the 1% level and are positive across all quartiles, indicating that the impact of green credit policy on increasing total factor productivity of businesses in various heavy polluters is always significant. Second, the regression coefficients are not equal at each quartile and the p-values are zero at various quartiles. This shows that the degree to which the application of green credit policy improves total factor productivity varies for various heavy polluters.

**TABLE 6:** Quantile regression results.

Variables	*lnTfp*	*lnTfp*	*lnTfp*	*lnTfp*
	(1)	(2)	(3)	(4)
*GCL*	0.521***	0.553***	0.532***	0.379***
	(19.65)	(24.30)	(23.77)	(12.17)
*lnAge*	0.189***	0.308***	0.451***	0.533***
	(4.68)	(8.90)	(13.25)	(11.24)
*lnCash*	–0.015	–0.007	–0.001	0.014
	(–0.96)	(–0.50)	(–0.10)	(0.75)
*lnRoa*	0.364***	0.335***	0.339***	0.288***
	(10.47)	(11.26)	(11.59)	(7.06)
*lnTang*	0.503***	0.341***	0.452***	0.441***
	(4.04)	(3.19)	(4.30)	(3.02)
*lnLev*	0.346***	0.409***	0.481***	0.563***
	(10.37)	(14.29)	(17.10)	(14.39)
*lnRoe*	–0.030	–0.007	–0.032	–0.012
	(–1.28)	(–0.34)	(–1.64)	(–0.45)
*Constant*	14.502***	14.562***	14.595***	14.992***
	(100.31)	(117.38)	(119.73)	(88.35)
*Quantile*	0.1	0.25	0.5	0.75
	P(q10 = q25 = q50 = q75) = 0.000			
*Observations*	7,014	7,014	7,014	7,014

t-statistics in parentheses, *** *p* < 0.01, ** *p* < 0.05, * *p* < 0.1.

### Mediation effect test

In order to investigate the mechanism underlying the impact of green credit policy on the total factor productivity of heavy polluting enterprises, this study employs a stepwise test to evaluate the mediating effect model of Equations (3)–(6), with the results shown in Table 7. The cost of debt is the mediating variable in Table 7 columns (1) to (3), which investigates whether green credit affects total factor productivity of businesses by influencing the cost of debt. Column 1 investigates the impact of green credit policy on business total factor productivity. Column (2) investigates the effect of green credit policy on firm debt costs and discovers that the estimated coefficient of green credit policy is positive and passes the 1% significance test, implying that green credit policy significantly raises firm debt costs. Column (3) investigates the effect of debt cost on total factor productivity further and discovers that the mediating variable cost of debt is significantly positive at the 1% level. By combining the results in column (2), we can conclude that green credit policies can significantly increase the cost of debt, and thus increase firm total factor productivity. The article employs Bootstrap to test the mediating effect, and the results show that debt cost has a partially mediating role in green credit and total factor productivity. The direct effect is 0.325, and the indirect effect is 0.048, accounting for approximately 12.9% of the total effect. This confirms the role of debt cost as a partial intermediary in green credit for enterprise transformation and upgrading, and hypothesis 2 is correct. The above studies show that green credit directs the rational allocation of resources among firms by increasing the cost of debt, and helps to bring into play the governance effect of financial resources, which in turn promotes the transformation and upgrading of heavy polluters.

**TABLE 7:** Stepwise regression results of intermediation effects.

Intermediate variables	*lnCdf*			*lnSubs*		
Variables	*lnTfp*	*lnCdf*	*lnTfp*	*lnTfp*	*lnSubs*	*lnTfp*
	(1)	(2)	(3)	(4)	(5)	(6)
*GCL*	0.373***	0.224***	0.325***	0.373***	0.499***	0.323***
	(17.50)	(5.06)	(17.00)	(17.50)	(10.23)	(15.46)
*lnCdf*			0.213***			
			(41.43)			
*lnSubs*						0.100***
						(19.63)
*lnAge*	0.354***	0.704***	0.204***	0.354***	0.679***	0.286***
	(10.92)	(10.44)	(6.97)	(10.92)	(9.15)	(9.02)
*lnCash*	0.013	–0.053**	0.024**	0.013	–0.010	0.014
	(1.00)	(–2.03)	(2.12)	(1.00)	(–0.33)	(1.11)
*lnRoa*	0.302***	0.554***	0.184***	0.302***	0.342***	0.268***
	(10.85)	(9.55)	(7.33)	(10.85)	(5.35)	(9.87)
*lnTang*	0.542***	0.374*	0.462***	0.542***	–0.217	0.564***
	(5.42)	(1.80)	(5.16)	(5.42)	(–0.95)	(5.79)
*lnLev*	0.469***	1.434***	0.164***	0.469***	0.529***	0.417***
	(17.52)	(25.73)	(6.52)	(17.52)	(8.62)	(15.89)
*lnRoe*	–0.016	–0.415***	0.073***	–0.016	–0.124***	–0.003
	(–0.83)	(–10.64)	(4.30)	(–0.83)	(–2.89)	(–0.18)
*Constant*	15.055***	19.634***	10.871***	15.055***	14.960***	13.564***
	(129.71)	(81.26)	(74.98)	(129.71)	(56.26)	(99.61)
Bootstrap test(ind-eff) Bootstrap test(dir-eff)	0.048 (0.000***)			0.05 (0.000***)		
	0.325(0.000***)			0.323 (0.000***)		
*Observations*	7,014	7,014	7,014	7,014	7,014	7,014
*R-squared*	0.171	0.294	0.335	0.171	0.068	0.215

t-statistics in parentheses, *** *p* < 0.01, ** *p* < 0.05, * *p* < 0.1.

Columns (4) to (6) of Table 7, the mediating variable is government subsidies, which is used to test the hypothesis that green credit affects firms’ total factor productivity by affecting government subsidies. Column (5) shows that the coefficient of green credit on government subsidies is significantly positive at the 1% level, indicating that green credit can significantly increase government subsidies; column (6) shows that the coefficient of government subsidies on total factor productivity of enterprises is significantly positive, and when combined with column (5), it can be concluded that green credit can significantly increase government subsidies and thus total factor productivity of enterprises. According to the results of their Bootstrap tests, government subsidies partially mediate the relationship between green credit and firms’ total factor productivity. The direct effect is 0.323, and the intermediary effect is 0.05, accounting for approximately 13.4% of the total effect. This supports the idea that government subsidies act as a partial intermediary in business transformation and upgrading, and hypothesis 3 is correct. In order to guide green credit to promote green operation of enterprises, the government needs to play its guiding role in planning and guiding industrial development through government subsidies, an important regulatory instrument, with a view to achieving harmonization of environmental and economic benefits.

### Moderating effect test

Moderating effects are those in which the independent variable influences the dependent variable by influencing the moderating variable, as opposed to mediating effects, which are those in which the independent variable influences the dependent variable by influencing the mediating variable. The purpose of this paper is to see if green credit can have a moderating effect on firm total factor productivity while already having a mediating effect on that productivity (Namazi and Namazi, 2016). As a result, this paper employs mediating variables as moderating variables directly. The regression estimation results of Equations (7) and (8) in this paper are shown in Table 8. The coefficient of the interaction term *GCL lnCdf* between green credit and the cost of debt in column (1) is positive and significant at the 1% level, indicating that the cost of debt is likely to positively regulate the impact of green credit on the total factor productivity of enterprises, i.e., as the cost of debt continues to increase, the role of green credit in promoting the green transformation of heavily polluting enterprises will strengthen. This may be due to the fact that green credit has imposed on clear provisions for banks and other financial institutions to carry out credit funding direction, methods and measures, and management and assessment, limiting the scale of financing and raising the cost of financing for heavily polluting enterprises. In the context of banks and other financial institutions practicing green development policies, the restrictions on debt financing for heavy polluters will become stricter and stricter, which will lead to the early transformation of heavy polluting enterprises from sloppy development to economical development. The coefficient of the interaction term *GCL lnSubs* between green credit and government subsidies in column (2) is significantly positive, indicating that government subsidies may positively moderate the impact of green credit on firms’ total factor productivity, i.e., with increasing government subsidies, green credit will promote the green transformation of heavy polluting firms. This demonstrates that the more widely green credit is implemented at this point, the more likely businesses are to invest the government subsidies they receive in green transformation and upgrading of their operations. Government subsidies have bolstered the role of green credit in promoting enterprise transformation and upgrading, while also increasing the effectiveness with which it is used. The benefits of green enterprise sustainability are bolstered by increased market-government synergy.

**TABLE 8:** Tests for moderating effects.

Variables	*lnTfp*	*lnTfp*
	(1)	(1)
*GCL*	0.373***	0.374***
	(17.53)	(17.57)
*GCL lnCdf*	0.057***	
	(5.61)	
*GCL lnSubs*		0.021**
		(2.05)
*lnAge*	0.344***	0.352***
	(10.63)	(10.86)
*lnCash*	0.009	0.012
	(0.74)	(0.92)
*lnRoa*	0.295***	0.301***
	(10.59)	(10.79)
*lnTang*	0.555***	0.541***
	(5.56)	(5.41)
*lnLev*	0.463***	0.470***
	(17.29)	(17.54)
*lnRoe*	–0.009	–0.014
	(–0.48)	(–0.74)
*Constant*	15.075***	15.059***
	(130.10)	(129.76)
*Observations*	7,014	7,014
*R-squared*	0.175	0.172

t-statistics in parentheses, *** *p* < 0.01, ** *p* < 0.05, * *p* < 0.1.

## Conclusion and policy implications

### Conclusion

There is no other way for China’s economy to transition to high-quality development than through enterprise transformation and upgrading. The implementation impact of green credit policy is extremely significant to support enterprise transformation and upgrading as it is a key measure to ease environmental resource constraints and realize green development strategy. Therefore, this study examines the effects of green credit policies on the green transformation and upgrading of heavy polluting enterprises using panel data of listed heavy polluting enterprises in China from 2007 to 2021 as samples. The influence mechanism is examined, and the following key conclusions are made: To begin with, following the implementation of the green credit policy, the credit threshold for businesses that produce a significant amount of pollution was significantly raised. This has a significant positive impact on the upgrading and greening of polluting businesses. Second, the intermediate effect model results show that government subsidies and corporate debt costs partially mediate the upgradation and transformation of highly polluting green credit enterprises. The moderating effect results also show that government subsidies and debt costs can both positively moderate the impact of green credit on enterprise transformation and upgrading, as well as the transformation and upgrading of highly polluting enterprises. The results of this paper show that green credit policy improves micro-governance efficiency through debt cost and government subsidies, and provides opportunities for enterprises to achieve a win-win situation of pollution control and efficiency improvement.

### Policy implications

Based on the above findings, the policy implications of this paper are as follows:

First, implementing green credit policy and obtaining bank credit support. China is going through a crucial stage of high-quality economic development, and the green credit policy will be a key factor in helping businesses go green. In order to achieve green transformation and upgrading, businesses should actively practice the concept of green development and grasp the macro-control direction during the transition period. As crucial components of the macro-control program of the Chinese government, banks and other financial institutions have a responsibility to manage their interactions with businesses in a professional manner and maintain open lines of communication. Therefore, they should clarify and refine the rules of green credit policy implementation, strictly follow the implementation of policy standards, provide detailed green credit audit results back to enterprises, clarify their shortcomings and improvement directions, and help them transform and upgrade in the green credit audit process. On the one hand, it gives businesses access to a wider range of financing options, diversifies the financing structure, and lessens the financial barriers to transforming their operations to be more environmentally friendly. On the other hand, expand the green gold project and guide enterprises to better obtain the support of relevant national policies.

Second, improve and adjust government subsidy policies to stimulate the green development of enterprises. As an important tool to correct market failures, government subsidies have a direct impact on the transformation and upgrading of enterprises. To ensure the efficient implementation of government subsidies, government subsidies should improve the corresponding monitoring mechanism and make the purpose of subsidy funds clear (Ding et al., 2022b). To implement differentiated subsidy strategies, maximize the impact of subsidy policies, government subsidies should be used in conjunction with actual industry development. Government subsidies should conform to the development strategies of different types of industries and improve the efficiency of government subsidies to promote the transformation and upgrading of heavy polluters. Simultaneously, policies should be altered to increase incentives for businesses to develop green output, limit unfair competition, and create a fair and healthy environment for enterprises to make efficient use of government subsidies.

Third, promote green governance and raise corporate environmental consciousness. To achieve carbon peaking and carbon neutrality, businesses should consider green credit policy as a development opportunity for transformation and upgrading, develop a green production and operation mode and optimize product structure. Actively use green financial products and green policies to advance green technological innovation and drive business transformation and upgrading. Perform well when corporations disclose environmental information and continue to improve the mechanism for disclosing environmental information. Reduce information asymmetry caused by a lack of green information, so that the production and operation of enterprises meet the criteria of green credit issuance and receive green credit support, thus realizing transformation and upgrading.

### Limitation and future research

This paper empirically tests the impact of green credit policy on the green transformation and upgrading of enterprises through corporate debt costs and government subsidies, and there may be other mediating variables that are not considered. In addition, the impact of green credit policies on other industries can be further tested, and these are questions worthy of continued exploration by the academic community in the future.

## Author contributions

HN: conceptualization, methodology, software, and writing—original draft preparation. XFZ and XHZ: data curation and writing—review and editing. ZL: writing—original draft preparation and data collection. YG: validation, formal analysis, investigation, resources, funding acquisition, and supervision. All authors have read and agreed to the published version of the manuscript.
